# Genetic Variants of *SIRT1* Gene Promoter in Type 2 Diabetes

**DOI:** 10.1155/2023/6919275

**Published:** 2023-01-28

**Authors:** Shuchao Pang, Zhengjun Zhang, Yu Zhou, Jie Zhang, Bo Yan

**Affiliations:** ^1^Shandong Provincial Sino-US Cooperation Research Center for Translational Medicine, Affiliated Hospital of Jining Medical University, Jining Medical University, Jining, Shandong 272029, China; ^2^Division of Endocrinology, Affiliated Hospital of Jining Medical University, Jining Medical University, Jining, Shandong 272029, China; ^3^Cardiovascular Center, Beijing Tongren Hospital, Capital Medical University, Dongcheng, Beijing 100730, China; ^4^Institute of Precision Medicine, Jining Medical University, Jining, Shandong 272067, China

## Abstract

Type 2 diabetes (T2D) is a highly heterogeneous and polygenic disease. To date, genetic causes and underlying mechanisms for T2D remain unclear. SIRT1, one member of highly conserved NAD-dependent class III deacetylases, has been implicated in many human diseases. Accumulating evidence indicates that SIRT1 is involved in insulin resistance and impaired pancreatic *β*-cell function, the two hallmarks of T2D. Thus, we speculated that altered SIRT1 levels, resulting from the genetic variants within its regulatory region of *SIRT1* gene, may contribute to the T2D development. In this study, the *SIRT1* gene promoter was genetically analyzed in T2D patients (*n* = 218) and healthy controls (*n* = 358). A total of 20 genetic variants, including 7 single-nucleotide polymorphisms (SNPs), were identified. Five heterozygous genetic variants (g.4114-15InsA, g.4801G > A, g.4816G > C, g.4934G > T, and g.4963_64Ins17bp) and one SNP (g.4198A > C (rs35706870)) were identified in T2D patients, but in none of the controls. The frequencies of two SNPs (g.4540A > G (rs3740051) (OR: 1.75, 95% CI: 1.24–2.47, *P* < 0.001 in dominant genetic model) and g.4821G > T (rs35995735)) (OR: 3.58, 95% CI: 1.94–6.60, *P* < 0.001 in dominant genetic model) were significantly higher in T2D patients. Further association and haplotype analyses confirmed that these two SNPs were strongly linked, contributing to the T2D (OR: 1.442, 95% CI: 1.080–1.927, *P* < 0.05). Moreover, most of the genetic variants identified in T2D were disease-specific. Taken together, the genetic variants within *SIRT1* gene promoter might contribute to the T2D development by altering SIRT1 levels. Underlying molecular mechanism needs to be further explored.

## 1. Introduction

Type 2 diabetes (T2D) is a highly heterogeneous and polygenic disease. Dysfunction of pancreatic *β* cells and insulin resistance in tissues are involved in the pathophysiology of T2D [1, 2]. Candidate gene association, linkage, and genome-wide association studies have identified large number of genetic loci and gene variants for T2D. A highly polygenic architecture of T2D has been established, which is dominated by common alleles with small and cumulative effects [3]. Although a small proportion of T2D cases can be explained, genetic causes and underlying molecular mechanisms of T2D remain largely unknown [4, 5]. Rare and low-frequency genetic variants that modulate *β*-cell mass and function may account for the missing inheritance for T2D [6].

Sirtuins are NAD-dependent protein deacetylases and broadly regulate many cellular processes, including cell fate determination, DNA damage repair, cellular protection, calorie restriction, and energy metabolism. Sirtuins have been implicated in age-related diseases, such as cancer, diabetes, and cardiovascular and neurodegenerative diseases [7–10]. There are seven members in the mammalian sirtuin family, SIRT1–7. SIRT1 gene is highly expressed in metabolically active tissues, including the liver, muscle, adipose tissue, heart, pancreas, and brain. SIRT1 regulates glucose and lipid metabolism, mitochondrial biogenesis, stress responses, inflammation, autophagy, circadian rhythms, and chromatin silencing [11]. In addition, SIRT1 is involved in the epigenetic regulation in the differentiation of the human stem cells [12, 13].

*SIRT1* has been involved in insulin resistance and impaired *β*-cell function, which are the hallmarks of T2D [14]. In experimental animals, SIRT1 regulates insulin secretion and protects pancreatic *β*-cells against toxic stresses [15, 16]. In mouse pancreatic beta cells, loss of SIRT1 leads to impaired glucose sensing and insulin secretion [17]. SIRT1 ameliorates insulin resistance by repressing protein tyrosine phosphatase 1B, a major negative regulator of insulin action [18]. In a T2D rat model, SIRT1 regulates glucose homeostasis and insulin sensitivity [19]. SIRT1 improves insulin sensitivity in skeletal muscle and liver [20, 21]. In cultured 3T3-L1 adipocytes and human adipose tissues, SIRT1 functions as a suppressor of inflammation, which is strongly associated with insulin resistance [22, 23]. Therefore, SIRT1 plays an important role in the T2D development.

The human *SIRT1* gene has been mapped to chromosome 10q21.3 [24]. The expression of the *SIRT1* gene is strictly controlled at transcription level. Hypermethylated in cancer 1 (HIC1), a transcriptional repressor, directly binds the *SIRT1* gene promoter and represses its transcription [25]. P53 has been shown to upregulate *SIRT1* gene expression by binding to a P53-binding element [26]. E2F1, a cell cycle and apoptosis regulator, induces the expression of the *SIRT1* gene [27]. Proinflammatory cytokine interferon gamma IFN-*γ* represses *SIRT1* gene expression [28]. In human pancreatic islet cells, SIRT1 is induced by gamma-aminobutyric acid (GABA), which protects pancreatic beta cells against apoptosis [29]. *SIRT1* gene is also regulated by extracellular-signal-regulated kinase 5 in leukemic Jurkat T cells [30].

Dysregulation of gene expression has been implicated in human diseases [31]. Variations in *SIRT1* gene expression levels have been associated with obesity and T2D [32–34]. *SIRT1* gene expression in circulating peripheral blood mononuclear cells is significantly associated with abdominal visceral fat accumulation [35]. In human adipose tissue, SIRT1 mRNA expression is significantly associated with energy expenditure and insulin sensitivity [36]. Therefore, we postulated that altered *SIRT1* gene expression levels, caused by the genetic variants within its regulatory regions, may contribute to the T2D development. Identification and subsequent functional analysis of genetic variants in *SIRT1* gene associated with T2D may provide a basis for manipulating *SIRT1* gene expression with genetic approaches or pharmaceutical chemicals as potential therapies for T2D patients. In the present study, the promoter region of the *SIRT1* gene was genetically analyzed in cohorts of T2D patients and controls.

## 2. Materials and Methods

### 2.1. Study Subjects

All T2D patients (*n* = 218, mean age: 52.44 years), including 123 males and 95 females, were recruited from the Division of Endocrinology, Affiliated Hospital of Jining Medical University, Jining Medical University, Jining, Shandong, China. T2D patients were diagnosed according to the American Diabetes Association guideline (2014) with fasting plasma glucose >7.0 mmol/L, 2-hour plasma glucose level >11.1 mmol/L, and glycated hemoglobin A1c >6.5%. Subjects with type 1 diabetes and other metabolic or endocrinological diseases were excluded from this study. The healthy controls (*n* = 358, mean age: 52.76 years), including 206 males and 152 females, were recruited from Physical Examination Center in the same hospital. Subjects with family history of T2D were excluded. This study was approved by the Human Ethics Committee of Affiliated Hospital of Jining Medical University. Informed consent was obtained from all participants. According to the power calculations for genetic association studies, more than 200 cases were included to eliminate the bias in different genetic models in this study [37–39].

### 2.2. Genetic Analysis

Peripheral leukocytes were isolated and genomic DNAs were extracted with DNeasy Blood and Tissue Kit (Qiagen, Valencia, CA, USA). The *SIRT1* gene promoter region, from −1051 bp upstream to +57 bp downstream to the transcription start site, was analyzed. Two overlapped DNA fragments, 551 bp and 592 bp, were amplified by PCR and directly sequenced. PCR primers were designed with the genomic sequence of human *SIRT1* gene (GenBank accession number: NG_050664.1). The 551 bp fragments (−1051 bp∼−501 bp) were generated with the PCR primers SIRT1-F1 (5′-GGAGTCACAGTGTGCCAGAA-3′) and SIRT1-R1 (5′-TTTCCCACTCTCCTCACACC-3′). The 592 bp fragment (−535 bp∼+57 bp) was generated with the PCR primers SIRT1-F2 (5′-AGGAGCTGTCAGAACGGTGT-3′) and SIRT1-R2 (5′-CCATCTTCCAACTGCCTCTC-3′). DNA sequencing was performed with 3730 DNA Analyzer (Applied Biosystems, Foster city, CA, USA). DNA sequences were aligned and compared with wild type *SIRT1* gene promoter. For heterozygous insertion or deletion genetic variants, the *SIRT1* gene promoter regions were subcloned into T-vector and directly sequenced. All genetic variants were further confirmed with PCR-generated DNA fragments and direct sequencing.

### 2.3. Statistical Analysis

Distributions of genetic variants were compared between T2D patients and controls using SPSS v13.0. The frequency of single-nucleotide polymorphisms (SNPs) in T2D and control groups was tested for deviation from Hardy–Weinberg equilibrium (HWE) by Fisher's test. Pearson chi-squared test was performed to evaluate the significant differences on allele and genotype frequencies between T2D patients and controls. The statistical power was generally set as 80% for determining the sample size that may yield the acceptable probability estimates. Odds ratio (OR) values and 95% confidence intervals (CIs) were measured using unconditional logistic regression analysis. The associations in different genetic models (codominant, dominant, over-dominant, and recessive) were analyzed with web-based software SNPStats. Linkage disequilibrium (LD) analysis and haplotype associations were conducted with Haploview software package (version 4.2) and SHEsis software platform. *P* < 0.05 was considered statistically significant.

## 3. Results

### 3.1. Genetic Variants Identified in T2D Patients and Controls

**Figure 1:**
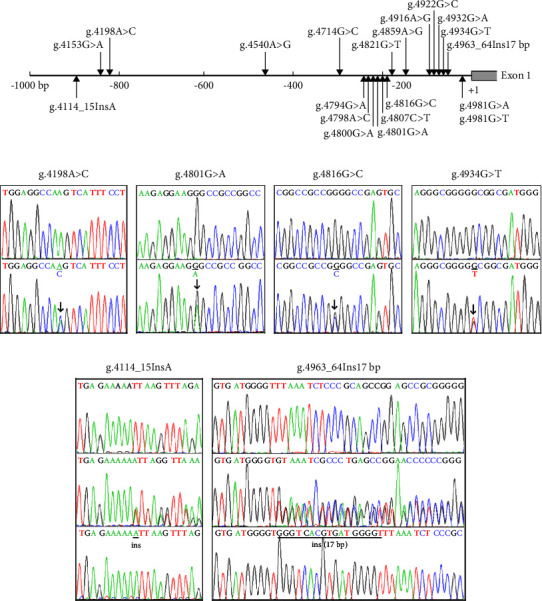
Genetic variants within the SIRT1 gene promoter in T2D patients and controls. (a) Schematic representation of the genetic variants in the SIRT1 gene promoter. The numbers represent the sequences of SIRT1gene promoter (Genbank accession number NG_050664.1). The transcription start site is at the position of 5008 of the first exon. (b) Sequencing chromatograms of the genetic variants which were only identified in T2D patients. These genetic variants were depicted in forward orientations. For genetic variants g.4198A > C (rs35706870), g.4801G > A, g.4816G > C, and g.4934G > T, top panels show the sequencing of wild type and bottom panels show the sequencing of heterozygous variants. For the insertion genetic variants, g.4114-15InsA and g.4963_64Ins17bp, top panels show the sequences of wild type, and middle panels show the sequences of heterozygous variants. To examine the exact insertion sequences, the SIRT1 gene promoter containing insertion genetic variants are subcloned into T-vectors, and then directly sequenced. The insertion sequences are shown in bottom panels. Heterozygous genetic variants are marked with solid arrows and the sequences of insertion genetic variants are underlined.

A total of 20 genetic variants, including 7 SNPs, were identified in this study population. Distribution and locations of the genetic variants are summarized in Table 1 and Figure 1(a). Five heterozygous genetic variants (g.4114-15InsA, g.4801G > A, g.4816G > C, g.4934G > T, and g.4963_64Ins17bp) and one SNP (g.4198A > C (rs35706870)) were identified in 16 T2D patients, but in none of the controls. More strikingly, the heterozygous insertion genetic variant (g.4963_64Ins17bp) was found in 11 T2D patients. The chromatograms of these genetic variants are depicted in Figure 1(b). Two SNPs (g.4540A > G (rs3740051) and g.4821G > T (rs35995735)) were more significantly frequent in T2D patients compared to controls (*P* < 0.01). In contrast, five heterozygous genetic variants (g.4153G > A, g.4794G > A, g.4800G > A, g.4859A > G, and g.4932G > A) and one SNP (g.4981G > A (rs575321146)) were only found in controls. Two SNPs (g.4798A > C (rs932658) and g.4922G > C (rs2394443)) were significantly more frequent in controls compared to T2D patients, respectively (*P* < 0.01 and *P* < 0.05). In addition, two heterozygous genetic variants (g.4714G > C and g.4807C > T) and two SNPs (g.4916A > G (rs3740053) and g.4981G > T (rs575321146)) were found in both T2D patients and controls with similar frequencies (*P* > 0.05).

### 3.2. Association between *SIRT1* Gene SNPs and T2D Risk

Seven SNPs were identified in this study, among which five SNPs were found with high frequency. Genotype distributions of the five SNPs in T2D patient and control groups (rs3740051, *P*=0.378; rs932658, *P*=0.263; rs35995735, *P*=0.095; rs3740053, *P*=0.542; rs2394443, *P*=0.263) were in HWE (*P* > 0.05). Distributions of genotypic and allelic frequencies of each SNP are shown in Table 2. The results showed that SNPs (g.4540A > G (rs3740051) and g.4821G > T (rs35995735)) were statistically associated with T2D.

Distributions of A/A, A/G, and G/G genotypes in SNP rs3740051 were 51.8%, 43.1%, and 5.0% in the T2D patient group and 65.4%, 31.8%, and 2.8% in the control group, respectively. There were significant associations between genotype frequency and distribution with the T2D patient group in codominant, dominant, and over-dominant models (*P*=0.005, *P* < 0.001, and *P*=0.006). The G allele frequency of rs3740051 was higher in the T2D patient group (26.6%) than in the control group (18.7%) (*P*=0.002).

Distributions of G/G, G/T, and T/T genotypes in SNP rs35995735 were 84.9%, 15.1%, and 0.0% in the T2D patient group and 95.3%, 4.5%, and 0.2% in the control group, respectively. There were significant associations between genotype frequency and distribution with the T2D patient group in codominant, dominant, and over-dominant models (*P* < 0.001, *P* < 0.001, and *P* < 0.001). The T allele frequency of rs35995735 was higher in the T2D patient group (7.6%) than in the control group (2.5%) (*P* < 0.001).

In addition, distributions of A/A, A/G, and G/G genotypes in SNP rs3740053 were 52.8%, 41.7%, and 5.5% in the T2D patient group and 61.7%, 34.4%, and 3.9% in the control group, respectively. There was a significant association between genotype frequency and distribution with the T2D patient group in the dominant model (*P*=0.34). The G allele frequency of rs3740053 was higher in the T2D patient group (26.4%) than in the control group (21.1%) (*P*=0.039).

### 3.3. Associations between Haplotypes and T2D Risk

**Figure 2:**
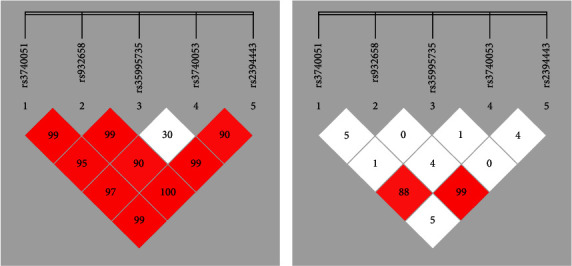
Linkage disequilibrium (LD) analysis for the five SNPs. The SNPs rs3740051, rs932658, rs35995735, rs3740053, and rs2394443 were analyzed. (a) Lewontin's standardized coefficient D' values. (b) Square of Pearson's correlation coefficient values (R2). Standard color schemes indicate different levels of LD.

To further analyze the association between haplotypes and T2D, we characterized the linkage disequilibrium (LD) of the *SIRT1* gene promoter SNPs in T2D patients and controls. D values and R2 values were examined with Haploview (version 4.2) and SHEsis (Figure 2). Two SNPs (rs35995735 and rs3740053) had no linkage, and all other SNPs showed strong linkage. Furthermore, R2 values showed a strong linkage between SNPs rs3740051 and rs3740053 as well as between SNPs rs932658 and rs2394443. These results further confirmed that SNPs rs3740051 and rs35995735 were associated with T2D.

The haplotypes of the five SNPs (rs3740051, rs932658, rs35995735, rs3740053, and rs2394443) and their frequencies in T2D patients and controls are shown in Table 3. The haplotypes G-A-G-G-G and A-A-T-A-G were associated with T2D (*P* < 0.05 and *P* < 0.001, respectively). The haplotype A-C-G-A-C provided protection from T2D (*P*<0.001). The most common haplotype A-A-G-A-G was not associated with T2D (*P* > 0.05).

### 3.4. Disease-Disease-Specificity of Genetic Variants in SIRT1 Gene Promoter

In previous studies, we have identified a number of genetic variants within *SIRT1* gene promoter in patients with acute myocardial infarction (AMI), Parkinson's disease (PD), and ventricular septal defects (VSD) [40–42]. In this study, we identified five genetic variants in T2D patients. These disease-related genetic variants are summarized in Table 4. Most genetic variants were disease-specific, including AMI (g.4198A > C, g.4324_25InsGCTG, g.4420_21InsG, and 0.4484G > C), PD (g.4614C > G, g.4794G > A, and g.4932G > A), VSD (g.4174A > G, g.4544A > T, and g.4552G > A), and T2D (g.4114-15InsA and g.4801G > A). In addition, genetic variants (g.4816G > C and g.4934G > T) were found in both AMI and T2D. Genetic variant (g.4963_64Ins17bp) was found in both VSD and T2D. Taken together, the genetic variants within *SIRT1* gene promoter had disease specificity.

## 4. Discussion

*SIRT1* gene mutations have been reported in type 1 diabetes [43]. Genetic variations in *SIRT1* gene have been related to the risk for obesity [44–46]. In a Dutch population, *SIRT1* gene SNPs are associated with prenatal famine exosure to influence the T2D risk [47]. In Pima Indians, an upstream variant (NC_000010.10: g.69635204T > A, rs10509291) and an intron variant (NC_000010.10: g.69651125A > G, rs7896005) in *SIRT1* gene are associated with reduced insulin secretion and increased risk for T2D [48]. In this study, we analyzed the proximal promoter region of the *SIRT1* gene and found six genetic variants in 7.3% (16/218) of T2D patients. The frequencies of two SNPs (g.4540A > G (rs3740051) and g.4821G > T (rs35995735)) were significantly higher in T2D patients compared to controls. Further genotypes analysis indicated that these two SNPs had strong linkage and were significantly associated with T2D in codominant, dominant, and over-dominant models. Collectively, these genetic variants and SNPs may abolish, create, or modify the binding sites for transcription factors within the *SIRT1* gene promoter, which then alter SIRT1 levels, contributing to the T2D development.

Many downstream targets of SIRT1 have been identified, including forkhead-box transcription factors (FOXOs), peroxisome proliferator-activated receptor *γ* (PPAR*γ*), PPAR*γ*-coactivator 1*α* (PGC-1*α*), myogenic differentiation 1, p53, and autophagy-related proteins. SIRT1 has been involved in insulin signaling by regulating insulin receptor substrate 2 and FOXO3 [49, 50]. In adipocytes, SIRT1 increases adiponectin gene expression in maintaining energy homeostasis [51]. In transgenic mice, adiponectin improves insulin sensitivity and acts against inflammation [52]. SIRT1 deacetylates FOXO1 in liver, adipose tissue, and pancreatic *β*-cells and protects *β*-cells against oxidative stress [53]. SIRT1 is involved in regulating inflammatory responses, gluconeogenesis, and levels of reactive oxygen species, which contribute to the insulin resistance [54, 55]. SIRT1 forms a complex with FOXA2 to regulate pancreas duodenum homeobox 1 (PDX1) gene, which is essential for pancreas development and *β*-cell formation [56]. In addition, SIRT1 induces autophagy by deacetylating autophagy-related (ATG) proteins, such as ATG5, ATG7, and LC3 (microtubule-associated protein 1 light chain 3 alpha) [57]. The crosstalk between SIRT1 and autophagy has been implicated in obesity and T2D [58]. Therefore, changed SIRT1 levels may affect pancreatic *β*-cell functions, insulin signaling, inflammation, autophagy, and other processes, contributing to the T2D development.

Genetic variants in *SIRT1* gene have been associated with many human diseases. T2D and coronary artery disease are closely linked. A number of genetic loci have been identified and shared in both diseases [59]. In patients with coronary artery disease, *SIRT1* gene expression levels are significantly decreased [60]. *SIRT1* gene SNPs have been associated with SIRT1 levels in patients with cardiovascular diseases [61]. In previous studies, we have identified several genetic variants within the *SIRT1* gene promoter in AMI patients, including g.4816G > C and g.4934G > T [40]. In this study, these two genetic variants were also found in T2D patients, providing further evidence that T2D and AMI shared common molecular mechanisms.

This study has limitations. *SIRT1* gene expression was not measured directly with clinical samples due to lack of sample availability. Moreover, the effects of the SNPs (g.4540A > G (rs3740051) and g.4821G > T (rs35995735)) on *SIRT1* gene expression need further study, as have been by previous studies [62, 63]. In addition, the impact of the SNPs on the onset and progression of T2D needs to be investigated.

In conclusion, we genetically analyzed the promoter region of *SIRT1* gene in T2D patients and controls. The genetic variants identified in T2D patients may contribute to the T2D development by changing SIRT1 levels. As natural and pharmacological compounds have been identified for regulating *SIRT1* gene expression, pharmacological targeting of *SIRT1* gene genetic variants may emerge as a novel therapy for T2D patients.

## Figures and Tables

**Table 1:** Genetic variants within the *SIRT1* gene promoter in T2D patients and controls.

Genetic variants	Genotypes	Location^1^	T2D (*n* = 218)	Controls (*n* = 358)	*P* value
g.4114-15InsA	−/A	−894 bp	1	0	—
g.4153G > A	GA	−855 bp	0	1	—
g.4198A > C (rs35706870)	AC	−810 bp	1	0	—
g.4540A > G (rs3740051)	AA	−468 bp	113	234	0.005
	AG		94	114	
	GG		11	10	
g.4714G > C	CG	−294 bp	1	2	1.000
g.4794G > A	GA	−214 bp	0	5	—
g.4798A > C (rs932658)	AA	−210 bp	167	230	0.007
	AC		48	118	
	CC		3	10	
g.4800G > A	GG	−208 bp	218	347	0.016
	GA		0	10	
	AA		0	1	
g.4801G > A	GA	−207 bp	1	0	—
g.4807C > T	CT	−201 bp	1	1	—
g.4816G > C	GC	−192 bp	1	0	—
g.4821G > T (rs35995735)	GG	−187 bp	185	341	<0.001
	GT		33	16	
	TT		0	1	
g.4859A > G	AG	−149 bp	0	1	—
g.4916A > G (rs3740053)	AA	−92 bp	115	221	0.099
	AG		91	123	
	GG		12	14	
g.4922G > C (rs2394443)	GG	−86 bp	167	230	0.007
	GC		48	118	
	CC		3	10	
g.4932G > A	GA	−76 bp	0	5	—
g.4934G > T	GT	−74 bp	1	0	—
g.4963_64Ins17bp	−/17 bp	−45 bp	11	0	—
g.4981G > A (rs575321146)	GA	−19 bp	0	1	—
g.4981G > T (rs575321146)	GT	−19 bp	1	1	—

^1^Locations of genetic variants upstream (−) to the transcription start site of SIRT1 gene at 5008 of NG_050664.1. *P* value: comparison of genotype frequencies between T2D patients and controls.

**Table 2:** Genotype distribution and allele frequencies of the SNPs in T2D patients and controls.

Genetic model	Genotypes	T2D (*n* = 218) (*n* (%))	Controls (*n* = 358) (*n* (%))	OR	*P* value
g.4540A > G (rs3740051)					
Codominant	AA	113 (51.8)	234 (65.4)	1.00	**0.005**
	AG	94 (43.1)	114 (31.8)	1.71 (1.20–2.43)	
	GG	11 (5.0)	10 (2.8)	2.28 (0.94–5.52)	
Dominant	AA	113 (51.8)	234 (65.4)	1.75 (1.24–2.47)	**0.001**
	AG + GG	105 (48.2)	124 (34.6)		
Recessive	AA + AG	207 (95.0)	348 (97.2)	1.85 (0.77–4.43)	0.162
	GG	11 (5.0)	10 (2.8)		
Over-dominant	AA + GG	124 (56.9)	244 (68.2)	1.62 (1.15–2.30)	**0.006**
	AG	94 (43.1)	114 (31.8)		
Allele	A	320 (73.4)	582 (81.3)	1.57 (1.19–2.09)	**0.002**
	G	116 (26.6)	134 (18.7)		
g.4798A > C (rs932658)					
Codominant	AA	167 (76.6)	230 (64.2)	1.00	**0.007**
	AC	48 (22.0)	118 (33.0)	0.56 (0.38–0.83)	
	CC	3 (1.4)	10 (2.8)	0.41 (0.11–1.52)	
Dominant	AA	167 (76.6)	230 (64.2)	0.55 (0.38–0.80)	**0.002**
	AC + CC	51 (23.4)	128 (35.8)		
Recessive	AA + AC	215 (98.6)	348 (97.2)	0.49 (0.13–1.78)	0.277
	CC	3 (1.4)	10 (2.8)		
Over-dominant	AA + CC	170 (78.0)	240 (67.0)	0.57 (0.39–0.85)	**0.005**
	AC	48 (22.0)	118 (33.0)		
Allele	A	382 (87.6)	578 (80.7)	0.59 (0.42–0.83)	**0.002**
	C	54 (12.4)	138 (19.3)		
g.4821G > T (rs35995735)					
Codominant	GG	185 (84.9)	341 (95.3)	1.00	**<0.001**
	GT	33 (15.1)	16 (4.5)	3.80 (2.04–7.09)	
	TT	0 (0.0)	1 (0.2)	—	
Dominant	GG	185 (84.9)	341 (95.3)	3.58 (1.94–6.60)	**<0.001**
	GT + TT	33 (15.1)	17 (4.7)		
Recessive	GG + GT	218 (100.0)	357 (99.8)	—	1.000
	TT	0 (0.0)	1 (0.2)		
Over-dominant	GG + TT	185 (84.9)	342 95.5)	3.81 (2.04–7.11)	**<0.001**
	GT	33 (15.1)	16 (4.5)		
Allele	G	403 (92.4)	698 (97.5)	3.18 (1.77–5.71)	**<0.001**
	T	33 (7.6)	18 (2.5)		
g.4916A > G (rs3740053)					
Codominant	AA	115 (52.8)	221 (61.7)	1.00	0.100
	AG	91 (41.7)	123 (34.4)	1.42 (1.00–2.02)	
	GG	12 (5.5)	14 (3.9)	1.65 (0.74–3.68)	
Dominant	AA	115 (52.8)	221 (61.7)	1.45 (1.03–2.03)	**0.034**
	AG + GG	103 (47.2)	137 (38.3)		
Recessive	AA + AG	206 (94.5)	344 (96.1)	1.43 (0.65–3.15)	0.371
	GG	12 (5.5)	14 (3.9)		
Over-dominant	AA + GG	127 (58.3)	235 (65.6)	1.37 (0.97–1.94)	0.075
	AG	91 (41.7)	123 (34.4)		
Allele	A	321 (73.6)	565 (78.9)	1.34 (1.02–1.78)	**0.039**
	G	115 (26.4)	151 (21.1)		
		(*n* (%))	(*n* (%))		
g.4922G > C (rs2394443)					
Codominant	GG	167 (76.6)	230 (64.2)	1.00	**0.007**
	GC	48 (22.0)	118 (33.0)	0.56 (0.38–0.83)	
	CC	3 (1.4)	10 (2.8)	0.41 (0.11–1.52)	
Dominant	GG	167 (76.6)	230 (64.2)	0.55 (0.38–0.80)	**0.002**
	GC + CC	51 (23.4)	128 (35.8)		
Recessive	GG + GC	215 (98.6)	348 (97.2)	0.49 (0.13–1.78)	0.277
	CC	3 (1.4)	10 (2.8)		
Over-dominant	GG + CC	170 (78.0)	240 (67.0)	0.57 (0.39–0.85)	**0.005**
	GC	48 (22.0)	118 (33.0)		
Allele	G	382 (87.6)	578 (80.7)	0.59 (0.42–0.83)	**0.002**
	C	54 (12.4)	138 (19.3)		

OR: odds ratio. *P* value: comparison of the genotype frequencies between T2D patients and controls. *P* values less than 0.05 were shown in bold.

**Table 3:** Haplotype analysis of the *SIRT1* gene SNPs in T2D patients and controls.

Haplotypes (SNPs (1–5))	T2D (*n* = 218) frequency (%)	Control (*n* = 358) frequency (%)	Chi2	Fisher's *P*	OR (95% CI)
A-A-G-A-G	240.14 (0.551)	427.06 (0.596)	2.628	>0.05	0.817 (0.639∼1.043)
A-C-G-A-C	47.31 (0.109)	136.93 (0.191)	14.060	<0.01	0.511 (0.358∼0.729)
G-A-G-G-G	107.29 (0.246)	131.93 (0.184)	6.186	<0.05	1.442 (1.080∼1.927)
A-A-T-A-G	30.53 (0.070)	0.00 (0.000)	51.357	<0.01	—

SNPs 1–5: rs3740051, rs932658, rs35995735, rs3740053, and rs2394443. Frequency: frequencies of haplotypes. OR: odds ratio.

**Table 4:** Summary of genetic variants within the *SIRT1* gene promoters in human diseases.

Genetic variants	Genotypes	Location^1^	AMI^2^ (*n* = 327)	PD^3^ (*n* = 97)	VSD^4^ (*n* = 333)	T2D^5^ (*n* = 218)
g.4114-15InsA	−/A	−894 bp	−	−	−	+
g.4174A > G	AG	−834 bp	−	−	+	−
g.4198A > C	AC	−810 bp	+	−	−	−
g.4324_25InsGCTG	−/GCTG	−684 bp	+	−	−	−
g.4420_21InsG	−/G	−588 bp	+	−	−	−
g.4484G > C	GC	−524 bp	+	−	−	−
g.4544A > T	AT	−464 bp	−	−	+	−
g.4552G > A	GA	−456 bp	−	−	+	−
g.4614C > G	CG	−394 bp	−	+	−	−
g.4794G > A	GA	−214 bp	−	+	−	−
g.4801G > A	GA	−207 bp	−	−	−	+
g.4816G > C	GC	−192 bp	+	−	−	+
g.4932G > A	GA	−76 bp	−	+	−	−
g.4934G > T	GT	−74 bp	+	−	−	+
g.4963_64Ins17bp	−/17 bp	−45 bp	−	−	+	+

^1^Locations of variants upstream (−) to the transcription start site of SIRT1 gene at 5008 of NG_050664.1; ^2^Cui et al. [40]; ^3^Zhang et al. [42]; ^4^Shan et al. [41]; ^5^this study. AMI, acute myocardial infarction; PD, Parkinson's disease; VSD, ventricular septal defects; T2D, type 2 diabetes.

## Data Availability

The data used to support the findings of this study are available from the corresponding author upon reasonable request.

## Abbreviations

AMI: Acute myocardial infarction
ATG5: Autophagy-related protein 5
ATG7: Autophagy-related protein 7
FOX1: Forkhead-box transcription factor 1
FOX3: Forkhead-box transcription factor 3
HIC1: Hypermethylated in cancer 1
LC3: Microtubule-associated protein 1 light chain 3 alpha
PD: Parkinson's disease
PGC-1*α*: PPAR*γ*-coactivator 1*α*
PPAR*γ*: Peroxisome proliferator-activated receptor *γ*
T2D: Type 2 diabetes
VSD: Ventricular septal defects.

## References

[1] Sameer A., Banday M., Nissar S. (2020). Pathophysiology of diabetes: an overview. *Avicenna J Med*.

[2] Kahn S. E., Cooper M. E., Del Prato S. (2014). Pathophysiology and treatment of type 2 diabetes: perspectives on the past, present, and future. *The Lancet*.

[3] Udler M. S. (2019). Type 2 diabetes: multiple genes, multiple diseases. *Current Diabetes Reports*.

[4] Langenberg C., Lotta L. A. (2018). Genomic insights into the causes of type 2 diabetes. *The Lancet*.

[5] Ling C., Bacos K., Rönn T. (2022). Epigenetics of type 2 diabetes mellitus and weight change - a tool for precision medicine?. *Nature Reviews Endocrinology*.

[6] Steinthorsdottir V., Thorleifsson G., Sulem P. (2014). Identification of low-frequency and rare sequence variants associated with elevated or reduced risk of type 2 diabetes. *Nature Genetics*.

[7] Longo V. D., Kennedy B. K. (2006). Sirtuins in aging and age-related disease. *Cell*.

[8] Giblin W., Skinner M. E., Lombard D. B. (2014). Sirtuins: guardians of mammalian healthspan. *Trends in Genetics*.

[9] Haigis M. C., Sinclair D. A. (2010). Mammalian sirtuins: biological insights and disease relevance. *Annual Review of Pathology: Mechanisms of Disease*.

[10] Hall J. A., Dominy J. E., Lee Y., Puigserver P. (2013). The sirtuin family’s role in aging and age-associated pathologies. *Journal of Clinical Investigation*.

[11] Houtkooper R. H., Pirinen E., Auwerx J. (2012). Sirtuins as regulators of metabolism and healthspan. *Nature Reviews Molecular Cell Biology*.

[12] Calvanese V., Lara E., Suárez-Alvarez B. (2010). Sirtuin 1 regulation of developmental genes during differentiation of stem cells. *Proceedings of the National Academy of Sciences of the United States of America*.

[13] Fang Y., Tang S., Li X. (2019). Sirtuins in metabolic and epigenetic regulation of stem cells. *Trends in Endocrinology and Metabolism*.

[14] Kitada M., Ogura Y., Monno I., Koya D. (2019). Sirtuins and type 2 diabetes: role in inflammation, oxidative stress, and mitochondrial function. *Frontiers in Endocrinology*.

[15] Bordone L., Motta M. C., Picard F. (2005). Sirt1 regulates insulin secretion by repressing UCP2 in pancreatic beta cells. *PLoS Biology*.

[16] Lee J. H., Song M. Y., Song E. K. (2009). Overexpression of SIRT1 protects pancreatic *β*-cells against cytokine toxicity by suppressing the nuclear factor-*κ*b signaling pathway. *Diabetes*.

[17] Luu L., Dai F. F., Prentice K. J. (2013). The loss of Sirt1 in mouse pancreatic beta cells impairs insulin secretion by disrupting glucose sensing. *Diabetologia*.

[18] Sun C., Zhang F., Ge X. (2007). SIRT1 improves insulin sensitivity under insulin-resistant conditions by repressing PTP1B. *Cell Metabolism*.

[19] Erion D. M., Yonemitsu S., Nie Y. (2009). SirT1 knockdown in liver decreases basal hepatic glucose production and increases hepatic insulin responsiveness in diabetic rats. *Proceedings of the National Academy of Sciences of the United States of America*.

[20] Li Y., Xu S., Giles A. (2011). Hepatic overexpression of SIRT1 in mice attenuates endoplasmic reticulum stress and insulin resistance in the liver. *The FASEB Journal*.

[21] Schenk S., McCurdy C. E., Philp A. (2011). Sirt1 enhances skeletal muscle insulin sensitivity in mice during caloric restriction. *Journal of Clinical Investigation*.

[22] Gillum M. P., Kotas M. E., Erion D. M. (2011). SirT1 regulates adipose tissue inflammation. *Diabetes*.

[23] Yoshizaki T., Milne J. C., Imamura T. (2009). SIRT1 exerts anti-inflammatory effects and improves insulin sensitivity in adipocytes. *Molecular and Cellular Biology*.

[24] Frye R. A. (1999). Characterization of five human cDNAs with homology to the yeast SIR2 gene: sir2-like proteins (sirtuins) metabolize NAD and may have protein ADP-ribosyltransferase activity. *Biochemical and Biophysical Research Communications*.

[25] Chen W. Y., Wang D. H., Yen R. C., Luo J., Gu W., Baylin S. B. (2005). Tumor suppressor HIC1 directly regulates SIRT1 to modulate p53-dependentDNA-damage responses. *Cell*.

[26] Naqvi A., Hoffman T. A., DeRicco J. (2010). A single-nucleotide variation in a p53-binding site affects nutrient-sensitive human SIRT1 expression. *Human Molecular Genetics*.

[27] Wang C., Chen L., Hou X. (2006). Interactions between E2F1 and SirT1 regulate apoptotic response to DNA damage. *Nature Cell Biology*.

[28] Li P., Zhao Y., Wu X. (2012). Interferon gamma (IFN-*γ*) disrupts energy expenditure and metabolic homeostasis by suppressing SIRT1 transcription. *Nucleic Acids Research*.

[29] Prud’homme G. J., Glinka Y., Udovyk O., Hasilo C., Paraskevas S., Wang Q. (2014). GABA protects pancreatic beta cells against apoptosis by increasing SIRT1 expression and activity. *Biochemical and Biophysical Research Communications*.

[30] Lopez-Royuela N., Rathore M. G., Allende-Vega N. (2014). Extracellular-signal-regulated kinase 5 modulates the antioxidant response by transcriptionally controlling Sirtuin 1 expression in leukemic cells. *The International Journal of Biochemistry & Cell Biology*.

[31] Lee T. I., Young R. A. (2013). Transcriptional regulation and its misregulation in disease. *Cell*.

[32] Clark S. J., Falchi M., Olsson B. (2012). Association of sirtuin 1 (SIRT1) gene SNPs and transcript expression levels with severe obesity. *Obesity*.

[33] Costa C. d S., Hammes T. O., Rohden F. (2010). SIRT1 transcription is decreased in visceral adipose tissue of morbidly obese patients with severe hepatic steatosis. *Obesity Surgery*.

[34] Song Y. S., Lee S. K., Jang Y. J. (2013). Association between low SIRT1 expression in visceral and subcutaneous adipose tissues and metabolic abnormalities in women with obesity and type 2 diabetes. *Diabetes Research and Clinical Practice*.

[35] Lee H., Chu S. H., Park J. Y., Park H. K., Im J. A., Lee J. W. (2013). Visceral adiposity is associated with SIRT1 expression in peripheral blood mononuclear cells: a pilot study. *Endocrine Journal*.

[36] Rutanen J., Yaluri N., Modi S. (2010). SIRT1 mRNA expression may be associated with energy expenditure and insulin sensitivity. *Diabetes*.

[37] Hong E. P., Park J. W. (2012). Sample size and statistical power calculation in genetic association studies. *Genomics Inform*.

[38] Ambrosius W. T., Lange E. M., Langefeld C. D. (2004). Power for genetic association studies with random allele frequencies and genotype distributions. *The American Journal of Human Genetics*.

[39] Kozlitina J., Xing C., Pertsemlidis A., Schucany W. R. (2010). Power of genetic association studies with fixed and random genotype frequencies. *Annals of Human Genetics*.

[40] Cui Y., Wang H., Chen H. (2012). Genetic analysis of the SIRT1 gene promoter in myocardial infarction. *Biochemical and Biophysical Research Communications*.

[41] Shan J., Pang S., Wanyan H., Xie W., Qin X., Yan B. (2012). Genetic analysis of the SIRT1 gene promoter in ventricular septal defects. *Biochemical and Biophysical Research Communications*.

[42] Zhang A., Wang H., Qin X., Pang S., Yan B. (2012). Genetic analysis of SIRT1 gene promoter in sporadic Parkinson’s disease. *Biochemical and Biophysical Research Communications*.

[43] Biason-Lauber A., Böni-Schnetzler M., Hubbard B. P. (2013). Identification of a SIRT1 mutation in a family with type 1 diabetes. *Cell Metabolism*.

[44] Kilic U., Gok O., Elibol-Can B. (2015). SIRT1 gene variants are related to risk of childhood obesity. *European Journal of Pediatrics*.

[45] Peeters A. V., Beckers S., Verrijken A. (2008). Association of SIRT1 gene variation with visceral obesity. *Human Genetics*.

[46] Zillikens M. C., van Meurs J. B., Rivadeneira F. (2009). SIRT1 genetic variation is related to BMI and risk of obesity. *Diabetes*.

[47] Botden I. P., Zillikens M. C., de Rooij S. R. (2012). Variants in the SIRT1 gene may affect diabetes risk in interaction with prenatal exposure to famine. *Diabetes Care*.

[48] Dong Y., Guo T., Traurig M. (2011). SIRT1 is associated with a decrease in acute insulin secretion and a sex specific increase in risk for type 2 diabetes in Pima Indians. *Molecular Genetics and Metabolism*.

[49] Brunet A., Sweeney L. B., Sturgill J. F. (2004). Stress-dependent regulation of FOXO transcription factors by the SIRT1 deacetylase. *Science*.

[50] Zhang J. (2007). The direct involvement of SirT1 in insulin-induced insulin receptor substrate-2 tyrosine phosphorylation. *Journal of Biological Chemistry*.

[51] Qiao L., Shao J. (2006). SIRT1 regulates adiponectin gene expression through Foxo1-C/enhancer-binding protein alpha transcriptional complex. *Journal of Biological Chemistry*.

[52] Luo N., Liu J., Chung B. H. (2010). Macrophage adiponectin expression improves insulin sensitivity and protects against inflammation and atherosclerosis. *Diabetes*.

[53] Kitamura T. (2013). The role of FOXO1 in *β*-cell failure and type 2 diabetes mellitus. *Nature Reviews Endocrinology*.

[54] Liang F., Kume S., Koya D. (2009). SIRT1 and insulin resistance. *Nature Reviews Endocrinology*.

[55] Purushotham A., Schug T. T., Xu Q., Surapureddi S., Guo X., Li X. (2009). Hepatocyte-specific deletion of SIRT1 alters fatty acid metabolism and results in hepatic steatosis and inflammation. *Cell Metabolism*.

[56] Wang R. H., Xu X., Kim H. S., Xiao Z., Deng C. X. (2013). SIRT1 deacetylates FOXA2 and is critical for Pdx1 transcription and *β*-cell formation. *International Journal of Biological Sciences*.

[57] Lee I. H., Cao L., Mostoslavsky R. (2008). A role for the NAD-dependent deacetylase Sirt1 in the regulation of autophagy. *Proceedings of the National Academy of Sciences of the United States of America*.

[58] Kim J. Y., Mondaca-Ruff D., Singh S., Wang Y. (2022). SIRT1 and autophagy: implications in endocrine disorders. *Frontiers in Endocrinology*.

[59] Goodarzi M. O., Rotter J. I. (2020). Genetics insights in the relationship between type 2 diabetes and coronary heart disease. *Circulation Research*.

[60] Hu Y., Wang L., Chen S. (2015). Association between the *SIRT1* mRNA Expression and Acute Coronary Syndrome. *Journal of Atherosclerosis and Thrombosis*.

[61] Kilic U., Gok O., Bacaksiz A., Izmirli M., Elibol-Can B., Uysal O. (2014). SIRT1 gene polymorphisms affect the protein expression in cardiovascular diseases. *PLoS One*.

[62] He J., Yuan L., Lin H. (2021). Genetic variants in m6A modification core genes are associated with glioma risk in Chinese children. *Molecular Therapy - Oncolytics*.

[63] Hua R. X., Fu W., Lin A. (2021). Role of FTO gene polymorphisms in Wilms tumor predisposition: a five-centercase-control study. *The Journal of Gene Medicine*.

